# Batch Experiments Demonstrating a Two-Stage Bacterial Process Coupling Methanotrophic and Heterotrophic Bacteria for 1-Alkene Production From Methane

**DOI:** 10.3389/fmicb.2022.874627

**Published:** 2022-05-19

**Authors:** Ramita Khanongnuch, Rahul Mangayil, Ville Santala, Anne Grethe Hestnes, Mette Marianne Svenning, Antti J. Rissanen

**Affiliations:** ^1^Materials Science and Environmental Engineering, Faculty of Engineering and Natural Sciences, Tampere University, Tampere, Finland; ^2^Department of Arctic and Marine Biology, UiT The Arctic University of Norway, Tromsø, Norway

**Keywords:** methane, methanotroph, organic acid production, 1-alkene, *Acinetobacter baylyi* ADP1

## Abstract

Methane (CH_4_) is a sustainable carbon feedstock for value-added chemical production in aerobic CH_4_-oxidizing bacteria (methanotrophs). Under substrate-limited (e.g., oxygen and nitrogen) conditions, CH_4_ oxidation results in the production of various short-chain organic acids and platform chemicals. These CH_4_-derived products could be broadened by utilizing them as feedstocks for heterotrophic bacteria. As a proof of concept, a two-stage system for CH_4_ abatement and 1-alkene production was developed in this study. Type I and Type II methanotrophs, *Methylobacter tundripaludum* SV96 and *Methylocystis rosea* SV97, respectively, were investigated in batch tests under different CH_4_ and air supplementation schemes. CH_4_ oxidation under either microaerobic or aerobic conditions induced the production of formate, acetate, succinate, and malate in *M. tundripaludum* SV96, accounting for 4.8–7.0% of consumed carbon from CH_4_ (C-CH_4_), while *M. rosea* SV97 produced the same compounds except for malate, and with lower efficiency than *M. tundripaludum* SV96, accounting for 0.7–1.8% of consumed C-CH_4_. For the first time, this study demonstrated the use of organic acid-rich spent media of methanotrophs cultivating engineered *Acinetobacter baylyi* ADP1 ‘*tesA-undA* cells for 1-alkene production. The highest yield of 1-undecene was obtained from the spent medium of *M. tundripaludum* SV96 at 68.9 ± 11.6 μmol mol C_substrate_^–1^. However, further large-scale studies on fermenters and their optimization are required to increase the production yields of organic acids in methanotrophs.

## Introduction

Methane (CH_4_) is the second most important greenhouse gas (GHG) after CO_2_, with a global warming potential (GWP) approximately 30 times higher than that of CO_2_ over a 100-year time horizon (IPCC, 2021). CH_4_ emissions from anthropogenic activities have been continuously increasing, accounting for approximately 60% of total CH_4_ emissions [based on top-down estimates reported by Saunois et al. (2020)]. Hence, stringent climate policy maintained over the next several decades, particularly regarding the use of CH_4_ as an energy source, is suggested to significantly reduce CH_4_ emissions (Harmsen et al., 2020). In environmental carbon flux, CH_4_-oxidizing bacteria are key regulators of CH_4_ abatement (Hanson and Hanson, 1996; Saarela et al., 2020). Due to its abundance and potential as a sustainable carbon feedstock, the development of biological processes for CH_4_ conversion to bio-based chemicals/liquid fuels is a promising and attractive research area (López et al., 2013; Sun et al., 2018; Liu et al., 2020).

**FIGURE 1:**
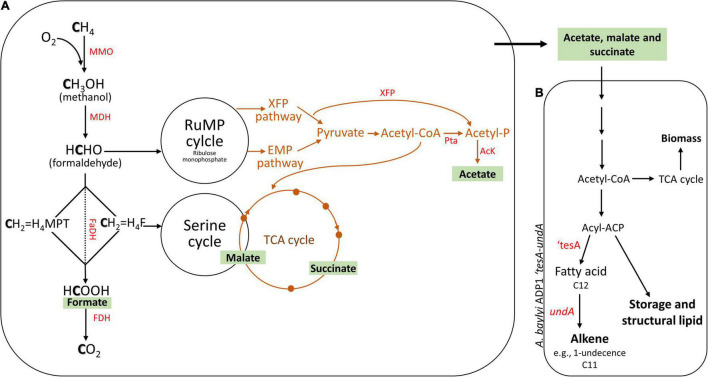
Principle of a two-stage bacterial process. The estimated pathway of the production of various organic acids could occur in methanotrophs **(A)**. Organic acid-rich spent medium was fed to the engineered *Acinetobacter baylyi* ADP1 for alkene production **(B)**. Aerobic CH_4_ oxidation pathway in Type I (RuMP cycle) and Type II (Serine cycle) methanotrophs (black arrows) and the possible pathway of CH_4_ oxidation under O_2_-limiting conditions *via* glycolysis-based CH_4_ fermentation mode (brown line). RuMP, ribulose monophosphate; EMP, Embden–Meyerhof–Parnas; TCA, tricarboxylic acid; MMO, methane monooxygenase; MDH, methanol dehydrogenase; FaDH, formaldehyde dehydrogenase; H_4_F, tetrahydrofolate pathway; H_4_MPT, tetrahydromethanopterin pathway; FDH, formate dehydrogenase; PTa, phosphate acetyltransferase; AcK, acetate kinase; XFP, phosphoketolase xylulose 5-phosphate/fructose 6-phosphate phosphoketolase; ‘*tesA*, thioesterase; *undA*, decarboxylase. Modified from Gilman et al. (2017) and Luo et al. (2019).

Aerobic CH_4_-oxidizing bacteria (methanotrophs) containing methane monooxygenases use CH_4_ as the sole carbon and energy source and oxygen as an electron acceptor (Hanson and Hanson, 1996; Khider et al., 2021). Methanotrophs are classified into gammaproteobacterial (Type I and Type X) and alphaproteobacterial (Type II) methanotrophs based on their different pathways for formaldehyde assimilation into biomass, which is the ribulose monophosphate (RuMP) and serine cycles, respectively (Kalyuzhnaya et al., 2015; Guerrero-Cruz et al., 2021). Along with these pathways, CH_4_ can be converted into various value-added products, including methanol, single-cell protein, ectoine, and soluble metabolites (Ge et al., 2014; Sheets et al., 2016; Strong et al., 2016; Cantera et al., 2018). In various environmental processes and biological systems, methanotrophs have been reported to support other bacteria by producing organic carbon sources from CH_4_, such as wastewater treatment (Costa et al., 2000; Cao et al., 2019) and heavy metal bioremediation (Lai et al., 2016; Karthikeyan et al., 2021). The pathways for the excretion of organic acids (e.g., formate, acetate, lactate, and succinate) have been observed in experiments and deciphered from the genomes of Type I methanotrophs during O_2_-limiting conditions including the Embden–Meyerhof–Parnas pathway and tricarboxylic acid (TCA) cycle (Figure 1; Kalyuzhnaya et al., 2013; Gilman et al., 2017). In addition to O_2_-limiting conditions, acetate production was also recently detected in liquid cultures of methanotrophs incubated under aerobic conditions (Lee et al., 2021; Takeuchi and Yoshioka, 2021). These studies also showed the potential of CH_4_-derived organic acids in biotechnological applications by cultivating Type I methanotrophs and heterotrophs in co-culture systems, where the heterotrophs utilize organic acids produced by methanotrophs (Lee et al., 2021; Takeuchi and Yoshioka, 2021). For example, acetate produced by *Methylocaldum marinum* S8 has been successfully used as a growth medium for *Cupriavidus necator* cultivation (Takeuchi and Yoshioka, 2021). In addition, a co-culture of *Methylococcus capsulatus* Bath and engineered *Escherichia coli* SBA01 has been demonstrated for mevalonate production (Lee et al., 2021).

*Acinetobacter baylyi* ADP1, a naturally competent gram-negative gammaproteobacteria, has been recently attracted interest in bioengineering. Recently, *A. baylyi* ADP1 was used for lignin valorization into long-chain alkyl esters and 1-alkene compounds (e.g., 1-undecene) by developing an engineered *A. baylyi* ADP1 (‘*tesA*-*undA*) strain (Luo et al., 2019; Salmela et al., 2020). As a platform chemical, 1-undecene can be used in the chemical synthesis of medium-chain poly-α-olefins (C33), which are commonly used as lubricants (Salmela et al., 2020). Furthermore, the ability of *A. baylyi* ADP1 to utilize fermentation by-products generated by other bacteria has been exploited to broaden the metabolic landscape of biological production processes (Salmela et al., 2018; Mangayil et al., 2019). Hence, *A. baylyi* ADP1, as well as its engineered strain, is a promising candidate for the bioconversion of CH_4_-derived organic acids. However, it is critical to identify a suitable methanotroph partner for such applications. For instance, whether Type I or Type II methanotrophs are good candidates for converting CH_4_ to organic acids for *A. baylyi* ADP1 is not known; to our knowledge, a comparison of organic acid production yields between Type I and Type II methanotrophs has not been previously reported. Nevertheless, CH_4_-derived compounds are limited to short carbon chain carbon compounds (C2–C6), which could be further used as feedstock for various heterotrophic bacteria and can be easily engineered to extend the range of CH_4_-derived value-added (platform) chemicals.

This study aimed to demonstrate the production of organic acids in both Type I and Type II methanotrophs and its application as a growth medium for an engineered *A. baylyi* ADP1 strain with the aim of high-value product formation. Thus, the growth and metabolite production profiles of Type I and Type II methanotrophs, *Methylobacter tundripaludum* SV96 and *Methylocystis rosea* SV97, respectively, were investigated under different gas supplementation schemes. Next, the possibility of using the spent media of methanotrophs as a growth substrate for the cultivation of wild-type *A. baylyi* ADP1 was tested. Finally, the synthesis of 1-alkenes from CH_4_ was demonstrated using a two-stage process with a methanotroph, that is, *M. tundripaludum* SV96 and *M. rosea* SV97, and an engineered *A. baylyi* ADP1 (‘*tesA*-*undA*) strain (Luo et al., 2019).

## Materials and Methods

### Strains and Cultivation Conditions

*M. tundripaludum* SV96 and *M. rosea* SV97 isolated from Arctic wetland soils in Norway (Wartiainen et al., 2006a,b) were used in this study. Methanotrophs were cultivated on nitrate mineral salt (NMS) medium (DSMZ medium 921; initial pH ∼6.80) with the addition of 1 mM lanthanum chloride (LaCl_3_). The pre-inoculum was grown in 120-ml serum bottles containing 10-ml NMS medium, 20% CH_4_, and 80% air in headspace and incubated statically at 20°C. All experiments were conducted under sterile conditions, and the serum bottles used for methanotroph cultivation were sealed with butyl rubber stoppers and capped with aluminum crimps.

Wild-type and engineered *A. baylyi* ADP1 strains (Luo et al., 2019) carrying the plasmid pBAV1C-*‘tesA*-*undA* (*A. baylyi* ADP1 ‘*tesA-undA*) were used to evaluate the growth on the methanotroph spent media. For the pre-inoculum, *A. baylyi* ADP1 cells were inoculated in 10-ml culture tubes containing LB medium (5 g L^–1^ yeast extract, 10 g L^–1^ tryptone, and 5 g L^–1^ NaCl) supplemented with 0.5% glucose and 25 μg mL^–1^ chloramphenicol (for *A. baylyi* ADP1 ‘*tesA-undA*). The inoculated tubes were aerobically grown overnight at 30°C and 300 rpm. For 1-undecene synthesis, *A. baylyi* ADP1 ‘*tesA-undA* was induced with 0.5 mM cyclohexanone (Luo et al., 2019).

### Evaluation of Organic Acid Production by *M. tundripaludum* SV96 and *M. rosea* SV97

**FIGURE 2:**
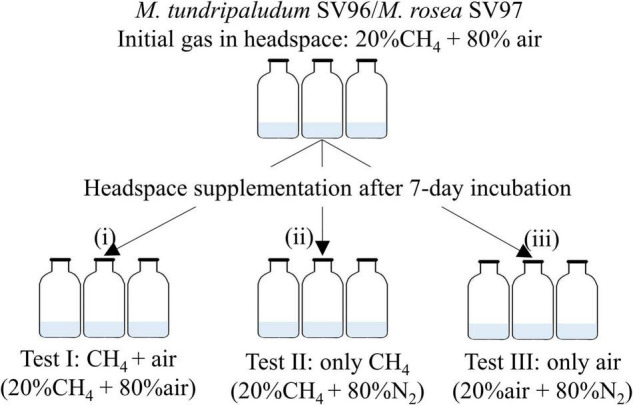
Experimental setup for methanotrophs under three different gas supplementation schemes applied on day 7 including test I: both CH_4_ and air added, test II only CH_4_ added, and test III only air added.

Batch tests were performed in 120-ml airtight serum bottles with a working volume of 15 ml of NMS medium. The tests were conducted in triplicates. The precultures of *M. tundripaludum* SV96 and *M. rosea* SV97 were inoculated at an initial optical density at 600 nm (OD_600nm_) of 0.02, and the growth, CH_4_ utilization, and organic acid profiles were monitored every 2 days for 14 days at 20°C under static conditions. Both methanotrophs were tested under the three different gas supplementation schemes (Figure 2). On day 0, the bottles were filled with 20% CH_4_ and 80% air into the headspace, accounting for the initial O_2_/CH_4_ molar ratio of ∼1.2, and incubated for 7 days. After the CH_4_ and O_2_ concentrations were depleted on day 7, the batch bottles were supplemented with three gas compositions into the headspace: test I: CH_4_ + air (20% CH_4_ and 80% air), test II: only CH_4_ (20% CH_4_ and 80% N_2_), and test III: only air (20% air and 80% N_2_). Bottles containing only NMS without bacterial cells were used as controls. The bottles were incubated for 7 days (days 8–14). Biomass growth (OD_600mm_), gas composition in the headspace, and organic acid accumulation in the liquid medium were monitored every 2 days.

### Cultivation of Wild-Type *A. baylyi* ADP1 in Spent Media of Different Methanotrophs

Prior to testing with an engineered *A. baylyi* ADP1, the capacity of *A. baylyi* ADP1 cells to utilize the spent media of methanotrophs was tested. To obtain methanotroph spent media, *M. tundripaludum* SV96 and *M. rosea* SV97 were cultivated in 500-ml airtight bottles with 60-ml NMS medium, as described previously (see section “Evaluation of Organic Acid Production by *M. tundripaludum* SV96 and *M. rosea* SV97”). To maximize the organic acid concentration, the bottle headspace was initially filled with 20% CH_4_ and 80% air and cultivated for 28 days under optimal conditions for *M. tundripaludum* SV96 and *M. rosea* SV97. The gaseous composition in the headspace and organic acid concentrations in the liquid medium were monitored twice a week.

After 28 days, the cultivation of methanotrophs was stopped, and spent media were used for *A. baylyi* ADP1 cultivation. The spent media was collected by centrifugation at 12,000 rpm for 10 min. The 50-ml supernatant of both methanotrophs was transferred to 250-ml sterile flasks and used for *A. baylyi* ADP1 cultivation. All tests were conducted in duplicate and incubated at 30°C and 300 rpm. After 4 h of cultivation, the liquid culture was collected to monitor the cell growth, organic acid concentration, and wax ester production. The spent media of methanotrophs without *A. baylyi* ADP1 were used as a contamination control (control 1), and *A. baylyi* ADP1 cells in NMS (fresh medium) were used to determine their background growth (control 2).

### Cultivation of *A. baylyi* ADP1 ‘*tesA-undA* Strain and 1-Alkene Synthesis in Spent Media of Different Methanotrophs

To obtain the methanotroph spent media, both *M. tundripaludum* SV96 and *M. rosea* SV97 were cultivated in 500-ml airtight bottles with 60-ml NMS medium, as described in section “Cultivation of Wild-Type *A. baylyi* ADP1 in Spent Media of Different Methanotrophs.” After 28 days, the spent media were collected and directly used for cultivation to identify any potential inhibitory effects on the growth of *A. baylyi* ADP1 cells imparted by the spent media supernatant (see section “Cultivation of Wild-Type *A. baylyi* ADP1 in Spent Media of Different Methanotrophs”). Thus, the original spent media, containing the methanotrophs and organic acids, devoid of any nutrient supplementation were employed as the growth medium for *A. baylyi* ADP1 cultivations in this experiment. The tests were conducted in quadruplicate in a sealed 20-ml glass tube containing 5 ml of the spent medium. Subsequently, *A. baylyi* ADP1 ‘*tesA*-*undA* cells (initial OD_600nm_, 0.02) were added to vials. After 1 h of incubation at 30°C and 300 rpm, 0.5 mM of cyclohexanone was added to the vials to induce 1-undecene synthesis and the vials were further incubated for 23 h at 30°C and 300 rpm. Liquid culture samples from *M. tundripaludum* SV96 and *M. rosea* SV97 cultivation without *A. baylyi* ADP1 *‘tesA-undA* were included as experimental controls. The growth of *A. baylyi* ADP1 ‘*tesA-undA* was estimated based on the difference in OD_600nm_ between the tests and controls after incubation for 24 h (ΔOD_600nm_).

### Analytical Methods

Liquid samples were filtered through a 0.2 μm membrane (Chromafil^®^ Xtra PET-20/25, Macherey-Nagel, Düren, Germany) prior to liquid metabolite analysis using a Shimadzu high-performance liquid chromatograph equipped with a Rezex RHM-Monosaccharide H^+^ column (Phenomenex, Torrance, CA, United States), as described by Okonkwo et al. (2018). The gas samples in the headspace (CH_4_, CO_2_, O_2_, and N_2_) were measured using a Shimadzu gas chromatograph GC-2014 equipped with a thermal conductivity detector and a Carboxen-1000 60/80 column (Agilent Technologies, Santa Clara, CA, United States). The oven temperature was held at 35°C for 3.75 min and then increased with the rate of 30°C min^–1^ until 150°C for 3 min. The injector and detector were 155 and 160°C, respectively. Helium was used as the carrier gas at 30 ml min^–1^.

1-undecene in the headspace was detected using solid phase microextraction gas chromatography–mass spectrometry, as previously described by Luo et al. (2019). Compounds were identified using the NIST/EPA/NIH Mass Spectral Library (NIST 05). Bacterial growth was measured as OD_600nm_ using an Ultrospec 500 pro spectrophotometer (Amersham Biosciences, United Kingdom) and as cell dry weight (CDW) using the gravimetric method. The conversion factor between CDW and OD_600nm_ obtained from the experimental measurements was 0.2914 g L^–1^ OD^–1^ (*R*^2^ = 0.9948) and 0.3015 g L^–1^ OD^–1^ (*R*^2^ = 0.9892) for *M. tundripaludum* SV96 and *M. rosea* SV97, respectively.

### Statistical Analysis

Statistical analysis was performed using Minitab 16.0 (United States). Significant differences in the obtained data sets (e.g., growth of the tested strains, gas concentrations and utilization, and concentrations and yields of the products produced by each strain) within the varied treatments were analyzed using one-way analysis of variance with Tukey’s multiple comparison tests at the 95% confidence interval, where *P*-value ≤ 0.05 was considered statistically significant.

## Results and Discussion

### CH_4_-Derived Organic Acid Production by *M. tundripaludum* SV96 and *M. rosea* SV97 in Different Gas Supplementation Tests

**FIGURE 3:**
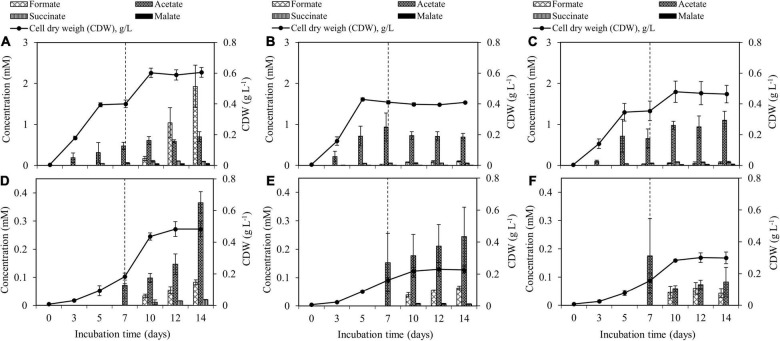
Accumulation of organic acid production and biomass production during the 14-day incubation of *M. tundripaludum* SV96 **(A–C)** and *M. rosea* SV97 **(D–F)** under three different gas supplementation schemes applied on day 7: (test I) both CH_4_ and air added **(A,D)**, (test II) only CH_4_ added **(B,E)**, and (test III) only air added **(C,F)**. Error bars indicate the standard deviation of triplicate samples.

Both *M. tundripaludum* SV96 and *M. rosea* SV97 used CH_4_ as the sole carbon and energy source and O_2_ as an electron acceptor. The biomass production of *M. tundripaludum* SV96 was greater than that of *M. rosea* SV97 in all tests (Figure 3). Highest biomass production of both methanotrophs was observed in the supplementation of both CH_4_ + air (test I) on day 14 at concentrations of 0.60 ± 0.03 and 0.48 ± 0.05 g CDW L^–1^ for *M. tundripaludum* SV96 and *M. rosea* SV97, respectively. In all tests, the growth yield of *M. rosea* SV97 was lower than that of *M. tundripaludum* SV96 (*P* < 0.05) because of the typical carbon assimilation pathway of Type I methanotrophs (RuMP), showing more efficient channeling of carbon from CH_4_ (C-CH_4_) to biomass than that of Type II methanotrophs (serine cycle) (Kalyuzhnaya et al., 2015).

The spent media of both methanotrophs contained similar organic acid compounds, including formate, acetate, and succinate, whereas malate was only present in the spent medium of *M. tundripaludum* SV96. However, the results suggest that *M. tundripaludum* SV96 is more efficient and promising for CH_4_ conversion into organic acids than *M. rosea* SV97. Furthermore, the concentration and production yield of total organic acids present in the spent medium of *M. tundripaludum* SV96 were higher than those of *M. rosea* SV97 in all gas supplementation tests (*P* < 0.05) (Figure 3). The efficiency of C-CH_4_ conversion into organic acids was 4.8–7.0% and 0.7–1.8% (of consumed C-CH_4_) for *M. tundripaludum* SV96 and *M. rosea* SV97, respectively (Supplementary Table 1). This could be due to the different carbon assimilation pathways between the RuMP and serine pathways in Type I and Type II methanotrophs. The RuMP pathway efficiently links to the glycolytic pathway, where pyruvate is converted to organic acids, whereas the serine cycle of Type II methanotrophs has a high flux through acetyl-CoA to yield an intracellular storage compound such as polyhydroxybutyrate (PHB) under nutrient-deficient conditions (Kalyuzhnaya et al., 2015; Nguyen et al., 2021). PHB is also a native product of *M. rosea* SV97 (Wartiainen et al., 2006b). Regarding the production of organic acids in Type II methanotrophs, which has not been widely reported, Costa et al. (2000) observed methanotroph-driven CH_4_ conversion into acetate, which was subsequently consumed by heterotrophs in a denitrification bioreactor using CH_4_ as an electron donor. Vecherskaya et al. (2009) also observed succinate, acetate, and 2,3-butanediol excreted in the culture medium of the Type II methanotroph *Methylocystis parvus* during CH_4_ oxidation under microaerobic and anaerobic conditions. Based on the ^13^C analysis in their study, these organic acids were likely from PHB degradation under microaerobic conditions (5–10% O_2_) (Vecherskaya et al., 2009).

**FIGURE 4:**
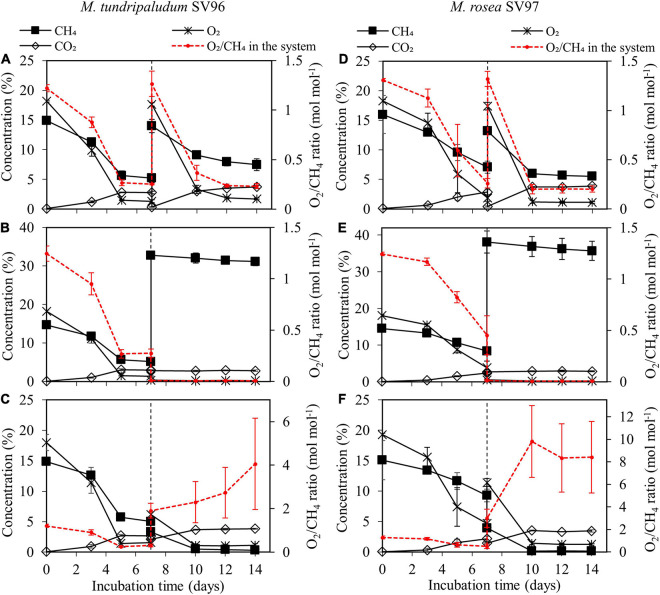
Gas compositions (CH_4_, O_2_, and CO_2_) in headspace during the 14-day incubation of *M. tundripaludum* SV96 (left column) and *M. rosea* SV97 (right column) under three different gas supplementation schemes applied on day 7: (test I) both CH_4_ and air added **(A,D)**, (test II) only CH_4_ added **(B,E)**, and (test III) only air added **(C,F)**. Error bars indicate the standard deviation of triplicate samples.

**FIGURE 5:**
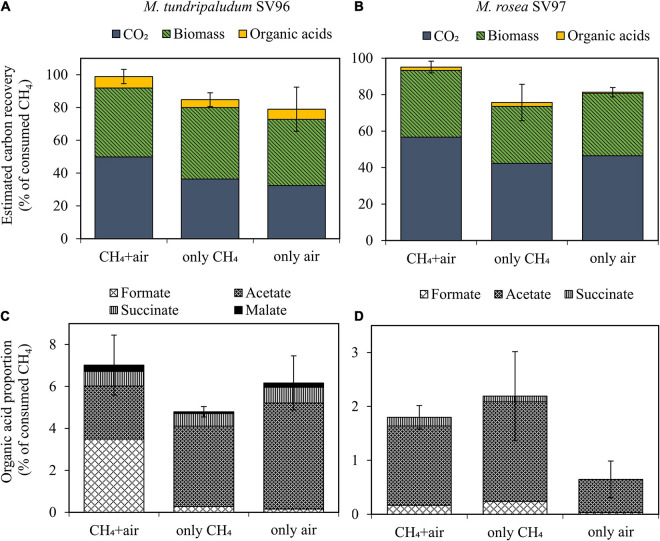
Carbon mass balance applied to CH_4_ oxidation to CO_2_, biomass, and organic acids **(A,B)** and distribution of each organic acid compound **(C,D)** of *M. tundripaludum* SV96 (left) and *M. rosea* SV97 (right) in three different gas supplementation tests for the 14-day incubation. Error bars indicate the standard deviation of sum of total yields in triplicate.

During the 14-day incubation period, organic acid concentrations gradually accumulated in the liquid culture of both methanotrophs in all gas supplementation tests, corresponding with the increase in their biomass production (Figure 3). On days 10–14 onward, three gas supplementation tests induced three different headspace conditions: microaerobic (O_2_-limited), anaerobic, and aerobic conditions. These conditions resulted in O_2_/CH_4_ molar ratios in the headspace of 0.2–0.3, <0.01, and 2–10 for the addition of CH_4_ + air (test I), CH_4_ (test II), and air (test III), respectively (Figure 4). Interestingly, the three gas supplementation tests in our study showed similar total organic acid production yields (Figures 5A,B). Lee et al. (2021) compared the production of organic acids in *M. capsulatus* Bath strain under various conditions, including aerobic, oxygen-limited, sulfur-limited, and nitrogen-limited conditions. The authors observed acetate production in all studied conditions, and nitrate-nitrogen limitation induced the highest acetate production (approximately 1.9 mmol-acetate g^–1^ CDW). Furthermore, previous studies on organic acid production by type I methanotrophs (Lee et al., 2021; Takeuchi and Yoshioka, 2021) have reported that acetate and formate are also produced under aerobic conditions but at lower concentrations than under O_2_-limited conditions. In our study, however, the three gas supplementation tests likely varied in the distribution of organic acids contained in the spent medium, particularly in *M. tundripaludum* SV96, a Type I methanotroph. Regarding organic acid production yields per consumed C-CH_4_, both formate (3.5%) and acetate (2.5%) were dominant in the test with CH_4_ + air supplementation (test I), whereas only acetate (5.1%) was dominant in the test with only air supplementation (test III) (Figures 5C,D). These results suggest that it is possible to target dominant organic acid compounds by the controlled feeding of CH_4_ and O_2_. The excretion of high formate concentration by *M. tundripaludum* SV96 during CH_4_ + air supplementation (test I) might be due to imbalanced growth during O_2_-limited conditions, which was previously reported in type I methanotrophs by Gilman et al. (2017). This also corresponded with the pH reduction observed in the test with CH_4_ + air supplementation (test I) (Supplementary Figure 1).

Compared to organic acid production in different Type I methanotroph species under O_2_-limited conditions, the *M. tundripaludum* SV96 used in our study showed comparable and higher organic acid yields per biomass production (CDW), that is, formate, acetate, and succinate (Table 1). These studies cultivated methanotrophs with initial headspace CH_4_ and O_2_ concentrations of 20 and 5%, respectively, to evaluate the organic acid production during microaerobic CH_4_ oxidation (Kalyuzhnaya et al., 2013; Gilman et al., 2015, 2017). However, in our study, *M. tundripaludum* SV96 was cultivated aerobically before allowing O_2_-limiting condition to occur. Further studies are required to determine whether prior cultivation under aerobic conditions enhances organic acid production under microaerobic conditions. In addition, the organic acid yields of *M. tundripaludum* SV96 obtained in our study (4.8–7.0% of consumed C-CH_4_) were higher than those of *M. capsulatus* Bath (<5% of consumed CH_4_) (Lee et al., 2021), whereas *M. alcaliphilum* 20Z enabled the convert 40–50% of the consumed CH_4_ into mostly acetate and formate under O_2_-limited conditions (20% CH_4_:5% O_2_) (Kalyuzhnaya et al., 2013).

**TABLE 1:** Production of organic acids and other metabolites obtained from different Type I methanotrophs cultivated under O_2_-limiting conditions.

Methanotrophs	Test mode	Volume	OD (CDW, g L^–1^)	Initial CH_4_:O_2_ (%)	Yield (mmol g CDW^–1^)				Note	References
					FM	AC	SC	ML		
*M. tundripaludum* SV96	Batch	15 mL in 120 mL vial	∼2 (0.6)	20:15 (10:3)^†^	3.20 ± 0.9	1.15 ± 0.17	0.16 ± 0.01	0.07 ± 0.01		Our study
*Methylomicrobium alcaliphilum* 20Z	Fed-batch/chemostat	1 L in 2 L chemostat	∼2 ± 0.2	20:5	1.53 ± 0.27	0.020	0.0025	ND	H_2_, lactate detected	Kalyuzhnaya et al. (2013)
*Methylomicrobium buryatense* 5GB1	Continuous	1 L in 2.5 L vessel	(0.45–0.46)	20:5	0.29–0.30	0.036–0.085	ND	ND	Lactate detected	Gilman et al. (2015)
	Fed-batch	1 L in 2.5 L vessel	(0.31–0.34)	10:5	0.40–0.67	0.096–0.123	ND	ND	Lactate detected	
*Methylomicrobium buryatense* 5GB1C (the aa3 cytochrome oxidase mutant strain)	Batch	1 L in 2.5 L vessel	(0.21)	20:5	1.22–3.00	0.081–0.086	ND	ND		Gilman et al. (2017)

*^†^The CH_4_:O_2_ in headspace when O_2_ limiting conditions occurring in the system. CDW, cell dry weight; ND, no data; FM, formate; AC, acetate; SC, succinate; ML, malate.*

### Utilization of Organic Acid-Rich Spent Media of Methanotrophs by *A. baylyi* ADP1

Wild-type *A. baylyi* ADP1 cells grew in methanotroph spent media containing formate, acetate, and succinate (and malate for *M. tundripaludum* SV96). During the 4-h incubation period, *A. baylyi* ADP1 cells incubated in the spent media from *M. tundripaludum* SV96 and *M. rosea* SV97 grew to an OD_600nm_ of 0.14 ± 0.01 and 0.12 ± 0.03, respectively (Supplementary Figure 2). In this test, *A. baylyi* ADP1 cells likely utilized acetate, succinate, and malate as carbon sources for biomass assimilation (Supplementary Figure 2). However, formate is not utilized by cells as a carbon source, but it is used to maintain the cellular redox balance (Kannisto et al., 2015). The presence of fatty acid fractions derived from *A. baylyi* ADP1 biomass on thin layer chromatography plates also indicated the growth of *A. baylyi* ADP1 (Supplementary Figure 3). Typically, *A. baylyi* ADP1 produces wax esters as carbon storage compounds that are often associated with growth (Mangayil et al., 2019). However, they were not detected in this study (Supplementary Figure 3). This may have been due to the rapid degradation of accumulated wax ester under carbon-limiting conditions. Under such conditions, *A. baylyi* ADP1 cells utilize stored carbon to maintain cellular activities (Fixter et al., 1986; Salmela et al., 2018). The use of the spent media of methanotrophs for wax production is a possible direction for further study, but this approach likely requires a higher quantity of organic acids.

### 1-Alkene Synthesis From Organic Acid-Rich Spent Media of Methanotrophs

**FIGURE 6:**
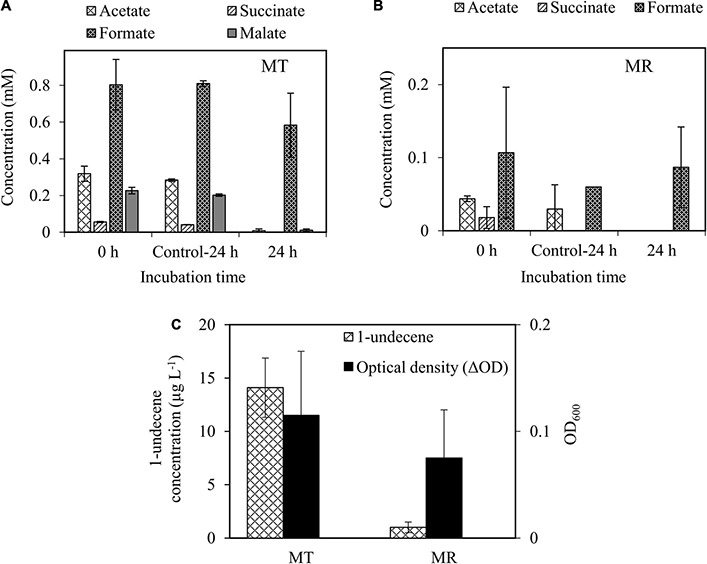
Organic acid utilization by *A. baylyi* ADP1 *‘tesA*-*undA* in spent media obtained from the cultivation of *M. tundripaludum* SV96 (MT) **(A)** and *M. rosea* SV97 (MR) **(B)** under O_2_-limiting conditions. Growth and 1-undecene production from *A. baylyi* ADP1 ‘*tesA*-*undA* after 24-h incubation **(C)**. ΔOD indicates the difference in OD_600nm_ of *A. baylyi* ADP1 ‘*tesA*-*undA* between 0 and 24 h. Error bars indicate the standard deviation of four replicate samples. The spent media from methanotrophs fermentation without *A. baylyi* ADP1 were used as control (Control-24 h).

After confirming the growth of wild-type *A. baylyi* ADP1 on the methanotroph spent media, organic acid-rich spent media were used as carbon sources for 1-alkene production by engineered *A. baylyi* ADP1 ‘*tesA-undA*. The results showed that *A. baylyi* ADP1 ‘*tesA-undA* completely utilized acetate (0.3 mM), succinate (0.05 mM), and malate (0.2 mM) present in the spent mxsedia of methanotrophs, except formate, similar to the wild-type ADP1 cells (Figure 6A). The production of 1-undecene from *M. tundripaludum* SV96 spent medium (14.1 ± 2.7 μg L^–1^) was higher than that from *M. rosea* SV97 cultivations (1.0 ± 0.5 μg L^–1^) (Figure 6C), corroborating with the organic acid concentrations (Figures 6A,B). Likewise, the growth of *A. baylyi* ADP1 ‘*tesA-undA* was higher in the spent medium from *M. tundripaludum* SV96 (0.115 ± 0.06 OD_600nm_) than from *M. rosea* SV97 (0.070 ± 0.054 OD_600nm_) (Figure 6C). Production of 1-undecene was not detected in the control cultures (Supplementary Figure 4). In addition, the biomass growth detected in the methanotroph spent media was from solely *A. baylyi* ADP1 ‘*tesA-undA* as both methanotrophs could not grow without the presence of CH_4_.

The obtained 1-undecene concentrations in this study (1.0 and 14.1 μg L^–1^) were lower than that previously reported for *A. baylyi* ADP1 ‘*tesA-undA* (Luo et al., 2019; Salmela et al., 2020). This phenomenon could be attributed to the higher substrate concentrations used in these studies. For example, Luo et al. (2019) developed a highly ferulate-tolerant *A. baylyi* ADP1 strain for 1-undecene production using adaptive laboratory evolution. The authors tested the use of a high concentration of ferulate (100 mM) as the sole carbon source and obtained a 1-undecene production titer of 72 ± 7.5 μg L^–1^, corresponding to a production yield of 1.0 μmol mol C_substrate_^–1^. In another study, Salmela et al. (2020) obtained 1-undecene concentration of up to ∼107 ± 8 μg L^–1^ from a two-stage system for 1-undecene production from cellulose which was converted into organic metabolites by *Clostridium cellulolyticum* (containing 5.2 mM glucose, 4.9 mM acetate, and 6.8 mM lactate). The authors reported the highest 1-undecene production yield of ∼35 μmol mol C_substrate_^–1^ using lactate as the substrate. Considering the 1-undecene production yield, the use of spent media of methanotrophs as a growth medium in our study was promising and comparable to those in previous studies, resulting in 68.9 ± 11.6 and 40.6 ± 19.8 μmol mol C_substrate_^–1^ for *M. tundripaludum* SV96 and *M. rosea* SV97, respectively. The carbon recovery obtained for 1-undecene production accounted for 0.065 and 0.045% of the total organic acids-carbon consumed by *M. tundripaludum* SV96 and *M. rosea* SV97, respectively (Supplementary Table 2). In our study, the spent media of methanotrophs likely did not contain intermediate compounds that were toxic to the growth and 1-undecene production of *A. baylyi* ADP1 ‘*tesA-undA*. Furthermore, the media can be directly used for cultivation without purification or additional downstream processing. However, in the future studies, the addition of some key macro/micronutrients should be evaluated, as it would benefit long-term process performance to increase 1-undecene concentration.

These results indicate that the spent media from microaerobic fermentation by methanotrophs are an excellent carbon source for heterotrophs. This two-stage bacterial process extends the range of CH_4_-derived products to the C11 compound (1-undecene). In further studies, the two-stage bacterial system will be scaled up and the process parameters will be optimized to be useful and competent for practical applications. In particular, the results obtained from our study show that different O_2_ and CH_4_ supplementation schemes in the headspace could lead to different concentrations and types of organic acids produced in the system. Furthermore, the contact time between methanotrophs and CH_4_ is important for maintaining active cells in fermenters and bioreactors (Guerrero-Cruz et al., 2021). In scale-up methanotroph cultivation, operational parameters such as O_2_ and CH_4_ inlet concentrations, dilution rates, and nitrogen sources should be optimized. The production of organic acids, particularly acetate and succinate, by methanotrophs has also been observed under aerobic conditions, suggesting potential strategies to develop a bioprocess system to co-cultivate methanotroph and *A. baylyi* ADP1 in a single system. In addition, the long-term effect of methanotroph cells in the spent media on the *A. baylyi* ADP1 growth and the 1-alkene production should be evaluated. For example, the spent media directly used as the *A. baylyi* ADP1 growth medium should be compared with the filtered and the centrifuged spent media prior to application.

## Conclusion

A two-step bioprocess setup was designed for the successful integration of microaerobic CH_4_ fermentation with aerobic synthesis. This study provides a proof of concept for integrating GHG utilization and platform chemical production: the application of organic acids produced by methanotrophs for 1-undecene (C11) production. A Type I methanotroph, *M. tundripaludum* SV96, showed a higher potential for organic acid production than a Type II methanotroph, *M. rosea* SV97, under aerobic and microaerobic conditions. The organic acid-rich spent media of methanotrophs could be directly used as a medium for the cultivation of wild-type *A. baylyi* ADP1 and engineered *A. baylyi* ADP1 ‘*tesA-undA* without additional downstream processes or purification. Acetate, succinate, and malate contained in the spent media were completely utilized by *A. baylyi* ADP1 ‘*tesA-undA* for 1-undecene production. The highest yield of 1-undecene was obtained from the spent medium of *M. tundripaludum* SV96 at 68.9 ± 11.6 μmol mol C_substrate_^–1^. However, the long-term effect of the methanotroph spent media on the 1-undecene production and the system scale-up requires further studies.

## Data Availability Statement

The original contributions presented in the study are included in the article/Supplementary Material, further inquiries can be directed to the corresponding author/s.

## Author Contributions

RK involved in conceptualization, writing—original draft, formal analysis, investigation, and visualization. RM involved in supervision, conceptualization, writing—review and editing, formal analysis, and investigation. VS and AH involved in methodology and writing—review and editing. MS involved in supervision, methodology, and writing—review and editing. AR involved in supervision, conceptualization, formal analysis, investigation, writing—review and editing, project administration, and funding acquisition. All authors contributed to the article and approved the submitted version.

## Conflict of Interest

The authors declare that the research was conducted in the absence of any commercial or financial relationships that could be construed as a potential conflict of interest.

## Publisher’s Note

All claims expressed in this article are solely those of the authors and do not necessarily represent those of their affiliated organizations, or those of the publisher, the editors and the reviewers. Any product that may be evaluated in this article, or claim that may be made by its manufacturer, is not guaranteed or endorsed by the publisher.
