# Supplementary material for: Batch Experiments Demonstrating a Two-Stage Bacterial Process Coupling Methanotrophic and Heterotrophic Bacteria for 1-Alkene Production From Methane
Source: Front Microbiol. 2022 May 19;13:874627. doi: 10.3389/fmicb.2022.874627 (PMC9162803; doi:10.3389/fmicb.2022.874627)
Supplement: Supplementary file 1 [file Data_Sheet_1.docx]

Supplementary Material

**Supplementary Table S1.** The carbon mass balance and distribution of consumed CH_4_-carbon after 14-day incubation of the experiments of *M. tundripaludum* SV96 (MT) and *M. rosea* SV97 (MR). All tests were incubated with both CH_4_ and air (20% CH_4_ and 80% air) in headspace on day 0 and three different gas supplementation schemes applied on day 7.

|  |  |  | *M. tundripaludum* SV96 | | | *M. rosea* SV97 | | |
| --- | --- | --- | --- | --- | --- | --- | --- | --- |
| Tests |  |  | **I: CH₄ + air** | **II: only CH₄** | **III: only air** | **I: CH₄ + air** | **II: only CH₄** | **III: only air** |
| Carbon mass (µmol) | Consumed CH_4_ | | 552.21 | 358.73 | 438.90 | 498.29 | 266.24 | 324.32 |
|  | Produced CO_2_ | | 275.13 | 130.20 | 141.56 | 281.89 | 112.38 | 150.81 |
|  | Biomass^†^ | | 231.91 | 156.17 | 177.27 | 182.44 | 83.04 | 111.10 |
|  | Produced organic acids | Formate(C1) | 19.28 | 0.99 | 0.67 | 0.83 | 0.63 | 0.11 |
|  |  | Acetate (C2) | 14.00 | 13.80 | 22.09 | 7.30 | 4.90 | 2.10 |
|  |  | Succinate (C4) | 3.79 | 2.12 | 3.26 | 0.80 | 0.27 | n.d. |
|  |  | Malate (C4) | 1.69 | 0.31 | 0.93 | n.d.^‡^ | n.d. | n.d. |
|  |  | Total organic acids | 38.77 | 17.22 | 26.95 | 8.93 | 5.81 | 1.50 |
|  | Total carbon products | | 545.80  ± 13.44 | 303.59  ± 8.61 | 345.79  ± 51.45 | 473.26  ± 23.74 | 201.22  ± 22.62 | 263.42  ± 28.30 |
| Carbon distribution of consumed CH_4_ (%) | Produced CO_2_ | | 49.8 | 36.4 | 32.4 | 56.7 | 42.3 | 46.5 |
|  | Biomass | | 42.0 | 43.6 | 40.4 | 36.6 | 31.2 | 34.2 |
|  | Produced organic acids | Formate(C1) | 3.5 | 0.3 | 0.2 | 0.2 | 0.2 | 0.0 |
|  |  | Acetate, (C2) | 2.5 | 3.8 | 5.1 | 1.5 | 1.8 | 0.6 |
|  |  | Succinate (C4) | 0.7 | 0.6 | 0.7 | 0.2 | 0.1 | 0.0 |
|  |  | Malate (C4) | 0.3 | 0.1 | 0.2 | 0.0 | 0.0 | 0.0 |
|  |  | Total organic acids | 7.0 | 4.8 | 6.2 | 1.8 | 2.2 | 0.7 |
| Carbon recovery (%) | |  | 98.9 ± 4.4 | 84.7 ± 4.2 | 79.0 ± 13.5 | 95.1 ± 3.2 | 75.7 ± 10.0 | 81.1 ± 2.6 |
| Organic acid yield (mmol g^-1^ CDW) | | Formate(C1) | 3.19 ± 0.89 | 0.24 ± 0.03 | 0.15 ± 0.07 | 0.17 ± 0.02 | 0.28 ± 0.04 | 0.13 ± 0.05 |
|  |  | Acetate, (C2) | 1.15 ± 0.17 | 1.68 ± 0.20 | 2.41 ± 0.60 | 0.76 ± 0.14 | 1.07 ± 0.35 | 0.28 ± 0.27 |
|  |  | Succinate (C4) | 0.16 ± 0.01 | 0.13 ± 0.01 | 0.17 ± 0.02 | 0.04 ± 0.00 | 0.03 ± 0.00 | n.d. |
|  |  | Malate (C4) | 0.07 ± 0.01 | 0.02 ± 0.00 | 0.05 ± 0.01 | n.d. | n.d. | n.d. |

Note: ^†^Chemical formula for biomass is from *Methylococcus capsulatus* = CH_2_O_0.5_N_0.27_ (25.78 g mol^-1^) (Popovic, 2019); ^‡^ n.d., not detected

Reference: Popovic, M. (2019). Thermodynamic properties of microorganisms: determination and analysis of enthalpy, entropy, and Gibbs free energy of biomass, cells and colonies of 32 microorganism species. *Heliyon*, *5*, e01950. https://doi.org/10.1016/j.heliyon.2019.e01950

**Supplementary Table S2.** Carbon mass balance applied to organic acid production by methanotrophs cultivated in a 500 mL vial and 1-undecene production from organic acid-rich spent medium of methanotrophs by *A. baylyi* ADP1 *tesA-undA* cultivated in a 5 mL vial.

|  |  | **MT** | **MR** | **MT** | **MR** |
| --- | --- | --- | --- | --- | --- |
|  |  | **Mass** | | **Carbon mass** | |
| **Carbon balance of organic acids production by MOBs (in 500 mL vial)** | **Substrate consumed by by MOBs** | |  |  |  |
|  |  | **µmol** | | **µmol** | |
|  | CH_4_ consumed by methanotrophs | 5218.43 | 5536.87 | 5218.43 | 5536.87 |
|  | **Products** |  |  |  |  |
|  | Produced CO_2_ | 1962 | 1668 | 1962 | 1668 |
|  | Produced biomass | 2261 | 3275 | 2261 | 3275 |
|  | Formate (CHO_2_^-^) | 80.32 | 10.68 | 80.32 | 10.68 |
|  | Acetate (C_2_H_3_O_2_^-^) | 31.87 | 4.38 | 63.75 | 8.77 |
|  | Succinate (C_4_H_4_O_4_^2-^) | 5.58 | 1.81 | 22.32 | 7.26 |
|  | Malate (C_4_H_4_O_5_^2-^) | 22.66 | 0.00 | 90.65 | 0 |
|  | Total |  |  | 4480.0 | 4969.3 |
|  |  |  |  | **% Carbon recovery** | |
|  | Recovery of C and e- for organic acids production from CH4 |  |  | 4.93 | 0.48 |
|  | Recovery of C and e- for CO2 production from CH4 |  |  | 37.60 | 30.12 |
|  | Recovery of C and e- for biomass production from CH4 |  |  | 43.32 | 59.14 |
|  | %Total carbon & electron recovery |  |  | 85.85 | 89.75 |
| **Carbon balance of 1-undecene production from organic acids by ADP1 (in 5 mL vial)** | **Substrate consumed by *A. baylyi tesA-undA*** | | |  |  |
|  |  | **µmol** | | **µmol** | |
|  | Acetate (C_2_H_3_O_2_^-^) | 1.55 | 0.22 | 2.32 | 0.44 |
|  | Succinate (C_4_H_4_O_4_^2-^) | 0.28 | 0.09 | 1.12 | 0.36 |
|  | Malate (C_4_H_4_O_5_^2-^) | 1.08 | - | 4.32 | - |
|  | Total |  |  | 7.76 | 0.80 |
|  | **Product** |  |  |  |  |
|  | 1-undecene (C_11_H_22_) | 0.0005 | 0.000033 | 0.00503 | 0.00036 |
|  | 1-undecene yield (µmol mol^-1^ Carbon substrate) | 58.86 | 40.63 |  |  |
|  |  |  |  | **% Carbon recovery** | |
|  | Carbon recovery for 1-undecene production from organic acids |  |  | 0.065 | 0.045 |
|  | Carbon recovery for 1-undecene production from consumed CH_4_ |  |  | 0.0001 | 0.00001 |

**Profiles of pH and optical density (OD) during cultivation of methanotrophs**

**
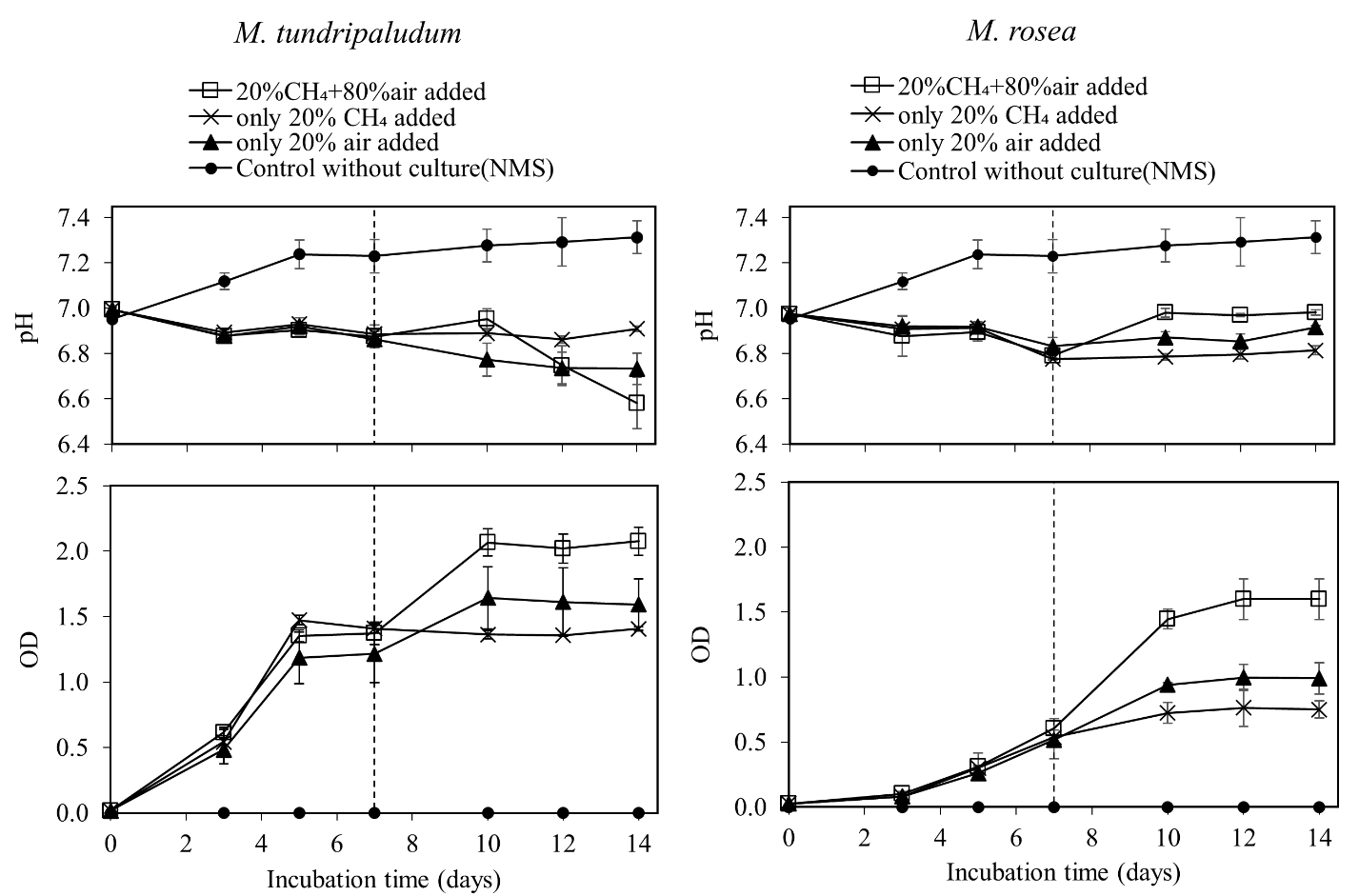
**

**Supplementary Figure S1.** Profiles of pH and OD during 14-day incubation of *M. tundripaludum* SV96 (**a**) and *M. rosea* SV97 (**b**) under three different gas supplementation schemes: (test I) both CH_4_ and air added, (test II) only air added, and (test III) only CH_4_ added. The error bars represent the standard deviations of triplicate samples.

**The growth of wild type *A. baylyi* ADP1 on spent medium of methanotrophs**


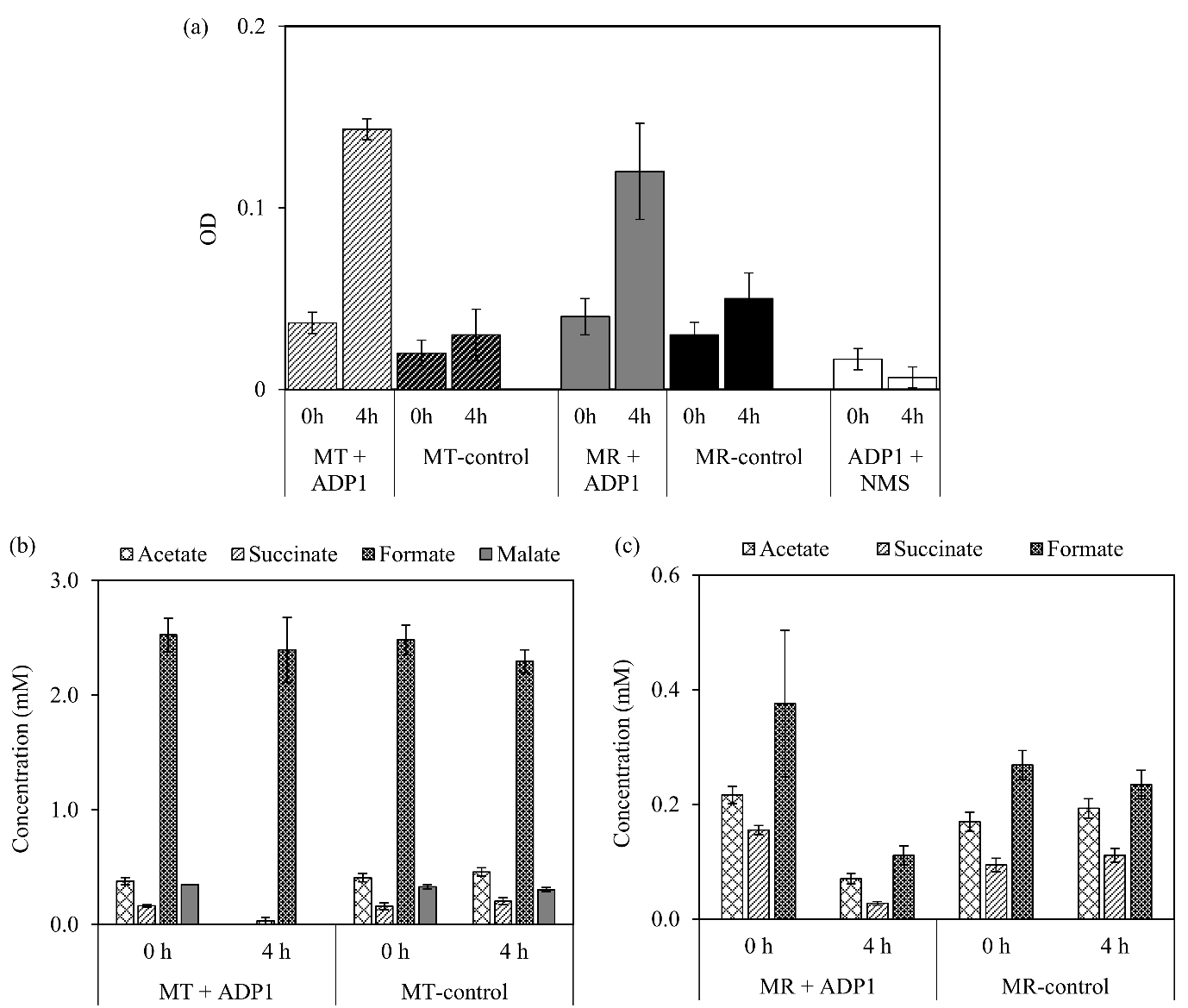


**Supplementary Figure S2.** Growth of wild type *A. baylyi* ADP1 after 4-h cultivation in the methanotroph spent media (**a**) and concentrations of organic acids contained in the spent media of *M. tundripaludum* SV96 (MT) (**b**) and *M. rosea* SV97 (MR) (**c**). Error bars indicate the standard deviation of duplicate samples. The incubations of spent media of MT and MR without *A. baylyi* ADP1 (MT- and MR-controls) and *A. baylyi* ADP1 with NMS fresh medium (ADP1 + NMS) were used as controls.

**Visualization of total lipid composition**


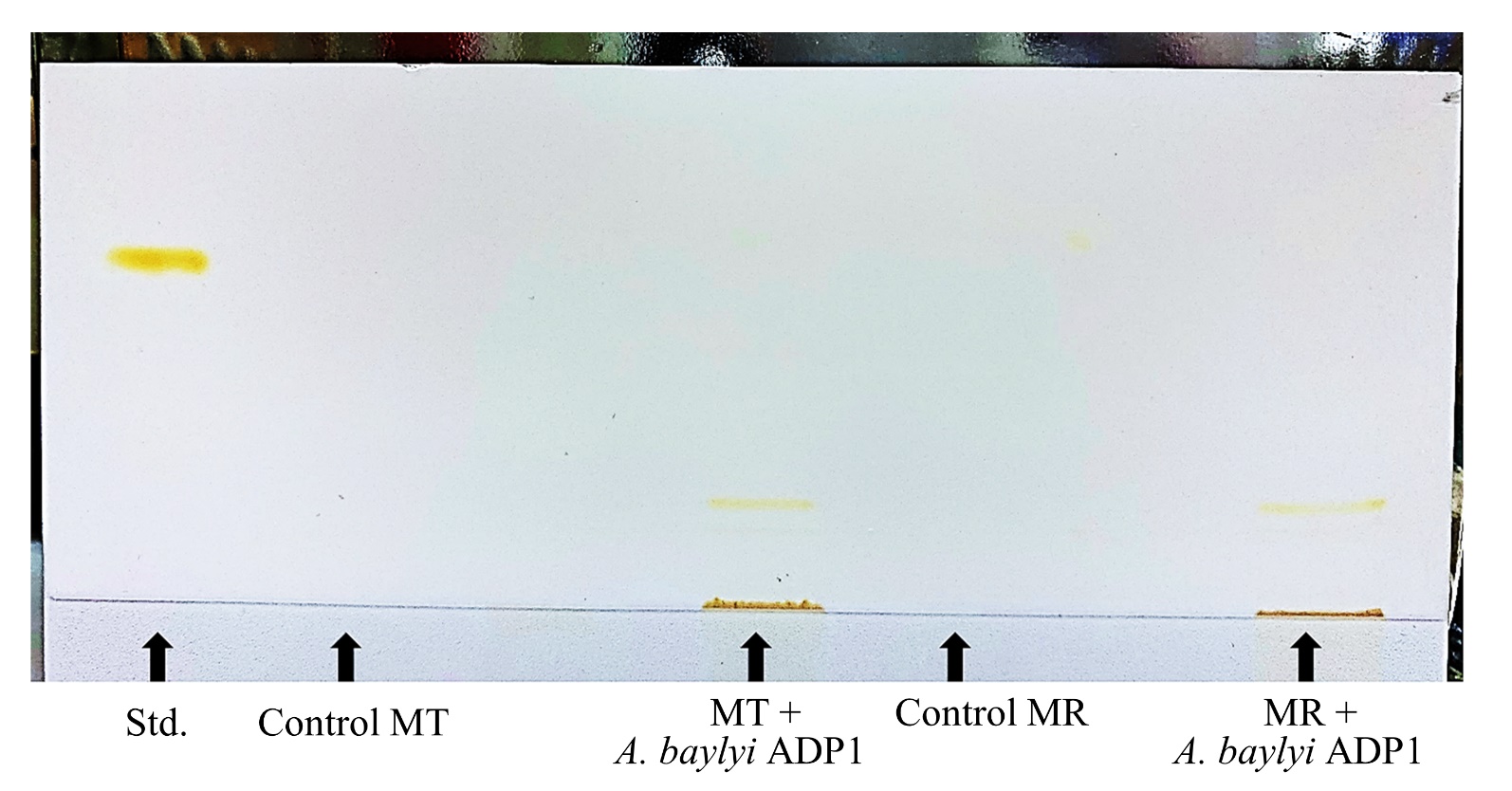


**Supplementary Figure S3.** The volumetric WE production in *A. baylyi* ADP1 cultivated in the spent media of *M. tundripaludum* SV96 (MT) and *M. rosea* SV97 (MR) were determined by thin layer chromatography analysis. Jojoba oil was used as the standard (Std.). The spent media without *A. baylyi* ADP1 were used as control1 (MT-control and MR-control). Wax ester production was detected by using lipid extraction and thin layer chromatography (TLC) analysis. Lipid extraction was done by using methanol-chloroform extraction described by Santala et al. (2011) and the obtained lipid phase layer was used for TLC analysis to visualize the total lipid composition. TLC analysis was done as described by Santala et al. (2011). Briefly, 60 µl of lipid phase of a sample was applied to TLC plate (10 × 20 cm HPTLC Silica Gel 60 F_254_ glass plates with 2.5 × 10 cm concentrating zone, Merck, USA) using n-hexane:diethyl ether:acetic acid of 90:15:1 as a mobile phase. The visualization was done by dyeing with iodine and Jojoba oil was used as a standard.

Reference: Santala, S., Efimova, E., Kivinen, V., Larjo, A., Aho, T., Karp, M., & Santala, V. (2011). Improved triacylglycerol production in *Acinetobacter baylyi* ADP1 by metabolic Engineering. *Microbial Cell Factories*, *10*, 36. https://doi.org/10.1186/1475-2859-10-36

**The present of 1-undecene in the cultivation detected by GC-MS**


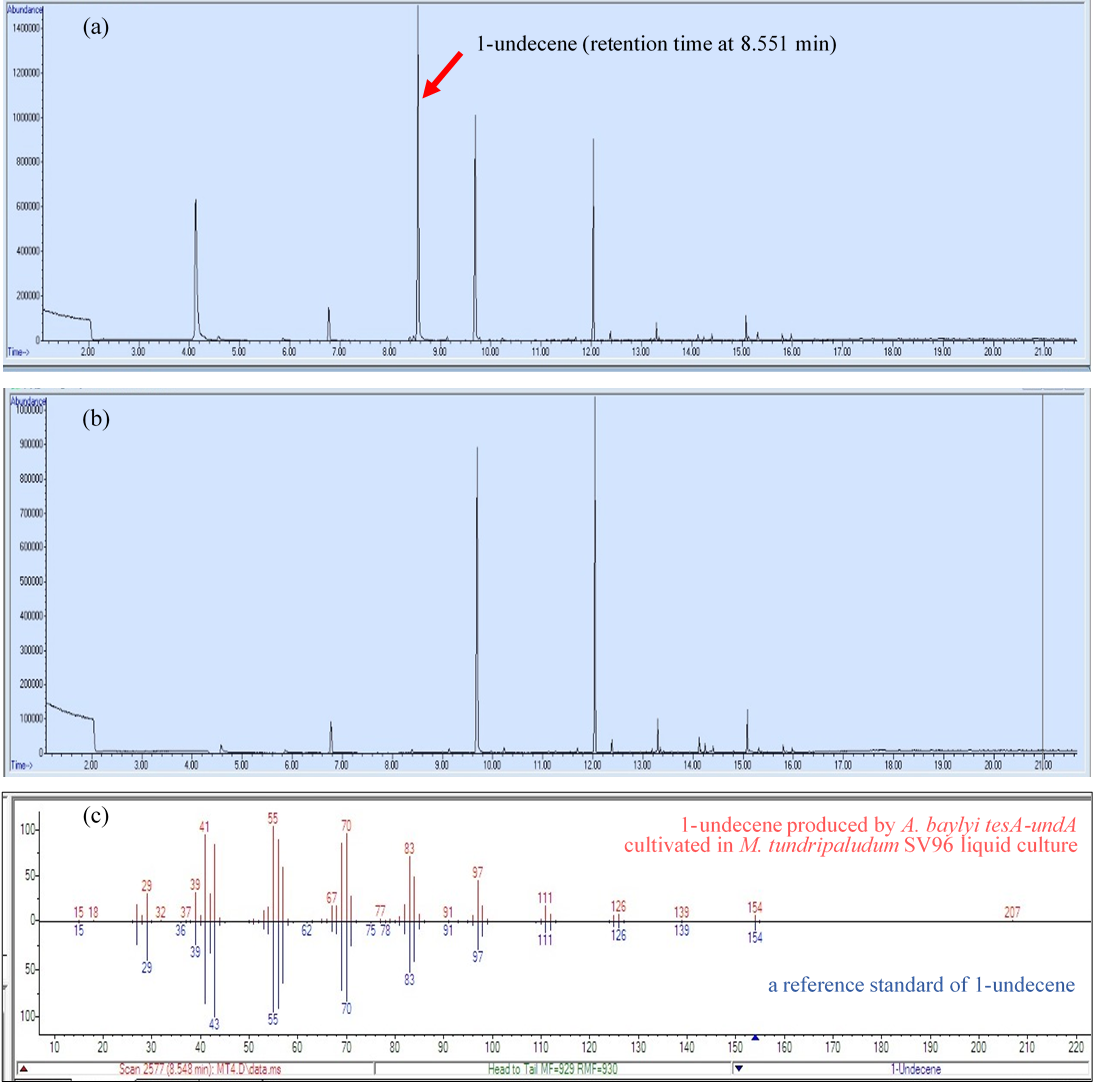


**Supplementary Figure S4.** GC-MS analysis for identifying 1-undecene in headspace of vials cultivating *A. baylyi tesA-undA* in *M. tundripaludum* SV96 spent medium (**a**) and the control cultivation of *M. tundripaludum* SV96 without *A. baylyi tesA-undA* (**b**). The reverse panel shows the reference standard of 1-undecene from NIST mass spectrometry data center (in blue) compared to the produced 1-undecene in the culture supernatant (in red) (**c**).
